# Life History Evaluation of *Ooencyrtus lucidus*, a Newly Described Egg Parasitoid of *Bagrada hilaris*

**DOI:** 10.3390/insects11050292

**Published:** 2020-05-09

**Authors:** Fatemeh Ganjisaffar, Thomas M. Perring

**Affiliations:** Department of Entomology, University of California, 900 University Ave., Riverside, CA 92521, USA; thomas.perring@ucr.edu

**Keywords:** bagrada bug, biological control, Encyrtidae, fecundity, life table, Pentatomidae

## Abstract

*Ooencyrtus lucidus* Triapitsyn & Ganjisaffar (Hymenoptera: Encyrtidae) was recently recovered from fresh sentinel eggs of *Bagrada hilaris* (Burmeister) (Hemiptera: Pentatomidae) in California. In the present study, life history traits of *O. lucidus* were studied at 26±0.5 °C, 40±10% RH, and a photoperiod of 14 L: 10 D hours. Results showed that 95.2% of the parasitized eggs yielded successful emergence of adults. The total immature developmental time was 14.27 and 13.95 days for females and males, respectively. The sex ratio of *O. lucidus* was 0.5 (females/offspring). Mated females laid on average 6.0 eggs per day and 222.7 eggs during their mean ovipositional period of 37.3 days. There was no significant difference in reproduction parameters between mated and non-mated females. The net reproductive rate ($R_{0}$) was 103.8 females/female/generation, the intrinsic rate of increase ($r_{m}$) was 0.171 females/female/day, the finite rate of increase (λ) was 1.187 population multiplication/day, the mean generation time (*T*) was 27.1 days, and the doubling time (*DT*) was 4.0 days. The highest longevity was observed in mated and non-mated females fed with honey, and they lived for 70.8 and 70.1 days, respectively. Providing females with host eggs in addition to honey, reduced their longevity by 24% in mated and 28% in non-mated individuals. Non-mated females and males deprived of honey only lived for 3.5 and 2.5 days after emergence, respectively. Our results indicate that *O. lucidus* has sufficient life history traits to make it a promising egg parasitoid for the biological control of *B. hilaris*.

## 1. Introduction

*Bagrada hilaris* (Burmeister) (Hemiptera: Pentatomidae), a stink bug native to Africa, Asia, and the Middle East [1,2], has invaded and colonized numerous regions of the world with serious damage to plants in the mustard family (Brassicaceae) [3,4]. These bugs are extremely damaging to young seedlings, but they also feed on leaves, stems, flowers, and the seeds of older plants [5,6]. *Bagrada hilaris* was accidentally introduced into California in 2008 [7], and by 2015 had spread significantly across the west coast of the United States [4,5,8,9,10,11]. In Mexico, *B. hilaris* was first reported in 2014 from the states of Saltillo and southeastern Coahuila causing economic damage to cole crops [12] and it reached the state of Guanajuato in 2017 [13]. This stink bug was introduced into Santiago, Chile in 2016 [14] and reports from Chile indicate that it is in the spreading stage of invasion [15,16].

Chemical applications have been the main approach against *B. hilaris* [4,17]. However, considerable efforts are underway to utilize biological control as part of the strategy to maintain pest populations below economic thresholds. These efforts include research on exotic natural enemies as classical biological control agents for *B. hilaris* [18,19] and investigations of native or introduced natural enemies associated with this pest in the field. To date, the adventive *Trissolcus hyalinipennis* Rajmohana & Narendran and the intentionally introduced *Trissolcus basalis* (Wollaston) (both Hymenoptera: Platygastroidea: Scelionidae) have been recovered from fresh sentinel eggs of *B. hilaris* in California [20]. In Mexico, two native species, *Telenomus podisi* Ashmead and *Gryon myrmecophilum* (Ashmead) (both Hymenoptera: Scelionidae), have been identified parasitizing *B. hilaris* eggs in the field [21]. In addition, *Idris elba* Talamas (Hymenoptera: Scelionidae), a primary parasitoid of spider eggs, was detected parasitizing *B. hilaris* eggs in Mexico [22]. Besides these scelionids, an encyrtid species was recovered from surveys in California in 2018, and this parasitoid has recently been described as *Ooencyrtus lucidus* Triapitsyn & Ganjisaffar (Hymenoptera: Encyrtidae) [23]. *Ooencyrtus lucidus* is the first native parasitoid reported to attack *B. hilaris* eggs in the United States. It appears that *O. lucidus* switched from parasitizing eggs of its native hosts, such as the green stink bug, *Chinavia hilaris* (Say) (Hemiptera: Pentatomidae), to *B. hilaris* eggs upon its invasion [23]. Such host switch has been reported for several parasitoids species [22,24,25]. In this study, the life history traits of this new species on *B. hilaris* eggs were determined under laboratory conditions.

## 2. Materials and Methods

### 2.1. The Host, *Bagrada hilaris*

*Bagrada hilaris* colonies were established with adult insects collected in Riverside, California in 2010 and were periodically supplemented with field collected insects to maintain genetic diversity. The bugs were maintained in tent-style insect cages (BugDorm^®^-2120, MegaView Science Co., Taipei, Taiwan) on greenhouse-grown Brassicaceae seedlings, *Lobularia maritima* (L.) (sweet alyssum), *Brassica oleracea* L. variety Italica (broccoli), *Brassica juncea* (L.) (green mustard), and *Brassica rapa* variety Japonica (mizuna), in a greenhouse at 30±5 °C with ambient humidity and light. To obtain eggs used in these studies, adult mating pairs were brought to an insectary room at the University of California, Riverside with 30±1 °C, 40–50% humidity, and 14 L: 10 D photoperiod and placed in round plastic containers (15 cm diameter × 6 cm height) (Durphy^®^ Packaging Co., Warminster, PA, USA) with two screen openings for ventilation. Paper towels were cut in circles and placed on the bottom of each container to provide an ovipositional substrate. Approximately 15 mating pairs were placed into each container (15–16 containers) and provided with organic broccoli florets as food every day. Adults were moved to new containers daily and the eggs laid within 24 h were used in the experiment or returned to the colony.

### 2.2. The Parasitoid, Ooencyrtus lucidus

The colony of *O. lucidus* was established from the wasps emerged from *B. hilaris* sentinel eggs parasitized in a squash field with mustard weeds in Riverside, California in 2018 [23]. This colony was maintained in an inverted plastic pantry container (11.4 cm diameter × 16.5 cm height) (Click Clack^®^ Co., Levin, New Zealand) in an incubator at 26±0.5 °C, 40 ± 10% humidity, and 14 L: 10 D photoperiod. The top was aerated by a 2.5-cm hole covered with a fine mesh. A 4-cm hole was made on the side of the container allowing access to the inside to provide the parasitoids with honey. This hole was plugged with a rubber stopper. Five to 6 lines of honey were streaked on a microscope slide which was placed inside the container and was replaced every 4–5 days. Thirty *B. hilaris* eggs (≤24 h old) were glued in 6 rows onto a 1.5 × 4 cm piece of card stock using the water-based Elmer’s^®^ glue. Since *O. lucidus* had been recovered from glued eggs of *B. hilaris* in the field [23], we were confident that glue did not inhibit parasitism of this parasitoid. To reduce superparasitism (parasitizing a host egg more than once by a single parasitoid species), the card was placed into a 2.5 × 9.5 cm glass vial (Fisher Scientific, Inc., Portsmouth, NH, USA) containing three to four 3-day-old mated females of *O. lucidus*. This vial also was streaked with honey and plugged with cotton. The egg card was replaced daily, and the parasitized egg card was moved to a new vial and kept until all wasps emerged. The wasps that emerged from these vials were either used in experiments or returned to the rearing container.

### 2.3. Experimental Procedure

Approximately 50 mating pairs of the newly emerged wasps (≤24 h old) were transferred into two glass vials (25 pairs in each) streaked with honey and kept in the aforementioned incubator for 3 days. *Bagrada hilaris* eggs were glued using Elmer’s^®^ glue on 10 cards (1.5 × 5 cm) made of a grid paper; each card had a 3 × 10 grid, containing 30 eggs. Each egg card was provided to three 3-day-old female wasps in a vial at a ratio of 1 wasp to 10 host eggs. After 24 h, the wasps were removed and eggs with one pedicel (non-superparasitized eggs) were cut out of the grid and placed in clear gel capsules size 00 (Capsuline^®^, Dania Beach, FL, USA). As is typical of many encyrtids and has been verified through host egg dissections in this study, each *O. lucidus* egg has a pedicel that protrudes from the host egg, serving as a respiratory tube for the developing larva [26]. The gel capsules were maintained in the same incubator at 26 °C and were checked daily until wasps emerged. The developmental time (egg to adult) and parasitism success (percentage of wasps emerged per parasitized eggs) were then recorded.

The emerged wasps (≤24 h old) then were used in the following treatments: (A) non-mated females and males were kept individually in separate vials and were provided only with honey, (B) a female and a male were kept together in the same vial and were provided only with honey, (C) a female and a male were kept together and were provided with both *B. hilaris* eggs and honey, (D) non-mated females were kept individually in vials and were provided with both *B. hilaris* eggs and honey, and (E) non-mated females and males were kept individually in vials with no eggs or honey. All wasps were held in 1.5 × 7.5 cm glass vials which were placed in separate trays for each treatment and they were held in the same incubator. The wasps were checked daily until they died. The initial number of replicates were 20 individuals for each sex in treatments A, B, D, and E, and 25 individuals of each sex in treatment C. Wasps that were stuck in honey were excluded from the analysis.

For treatments C and D, fourteen *B. hilaris* eggs (≤24 h old) were glued on 1 × 3.5 cm cards made of a 2 × 7 grid. The number of eggs provided was determined based on preliminary tests to be ad libitum. Every day, the wasps were provided with a new egg card, and the number of pedicels on each egg of the parasitoid-exposed egg card from the previous day was counted and the level of superparasitism also was determined. The egg card then was moved to a new vial and checked daily for wasp emergence for 30 days following oviposition. *Bagrada hilaris* nymphs that emerged from non-parasitized eggs were removed to prevent them from cannibalizing the remaining eggs. Although a low number of non-parasitized eggs died without hatching, we were not confident to associate that mortality with a factor induced by the wasps, such as host feeding, since the average mortality of *B. hilaris* eggs at 26 °C has been reported to be 11.7% [27]. Emerged wasps were sexed upon emergence and were transferred to the colony container. The parasitized eggs that did not hatch were dissected and the stage during which the mortality occurred was recorded. The adults that did not emerge were sexed and the numbers were included in the sex ratio calculation.

### 2.4. Statistical Analyses

For comparisons of developmental times and longevities between females and males, and pre-oviposition, oviposition, and post-oviposition periods between mated and non-mated females in treatments C and D, data first were tested for normality using the Shapiro-Wilk test (*p* < 0.05). Data then were analyzed using either a t-test or the nonparametric Wilcoxon-Mann-Whitney test based on the distribution of the data. The fecundity data and parasitism success rates between mated and non-mated females were discrete data that were also analyzed using the Wilcoxon-Mann-Whitney test. Longevity data for each sex were compared between all five treatments using one-way ANOVA followed by the nonparametric Dunn’s Kruskal-Wallis multiple comparison. All data were analyzed in R [28] with the alpha (α) significance threshold set at 0.05. A life table for treatment C was constructed using the survival and reproduction data [29]. Then, the Jackknife procedure [30] was used to calculate the population growth parameters and their mean and standard errors [31]. Mortality percentages at each stage were compared between mated and non-mated females using Chi-squared test (*p* < 0.05).

## 3. Results

**Figure 1:**
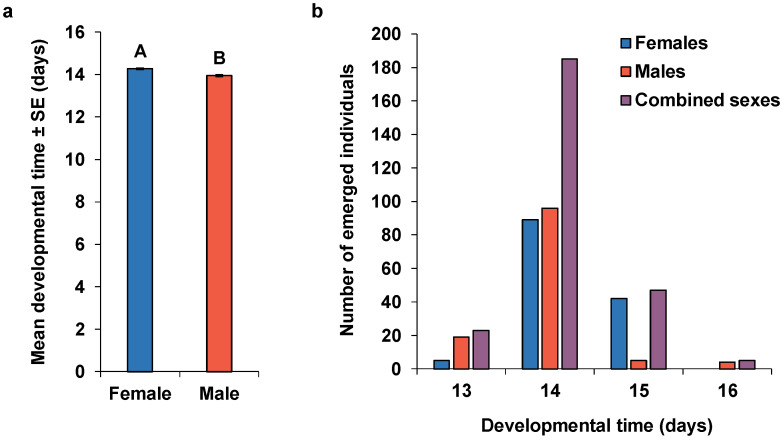
(**a**) The mean (±SE) developmental times of the two sexes. Bars accompanied by different letters are significantly different (Wilcoxon-Mann-Whitney test, *P* < 0.0001). (**b**) Distribution of developmental times in *Ooencyrtus lucidus* females (n = 136), males (n = 124), and combined sexes (n = 260).

Of the initial 300 *B. hilaris* eggs provided to *O. lucidus* females, 273 eggs with one pedicel were collected from which 260 wasps developed successfully, yielding an emergence success of 95.2%. *Ooencyrtus lucidus* females and males completed their development at 26 °C in 14.27 ± 0.04 and 13.95 ± 0.05 days, respectively, which were significantly different (*Z* = 5.22, *p* < 0.001) (Figure 1a). The first day of emergence was 13 days after oviposition for both sexes. On day 13, 3.7% of females and 15.3% of males emerged. By the next day, 69.1% of all female progeny and 92.7% of all male progeny emerged. Combining both sexes, 80.0% of the eggs developed to the adult stage in 14 days (Figure 1b).

**Figure 2:**
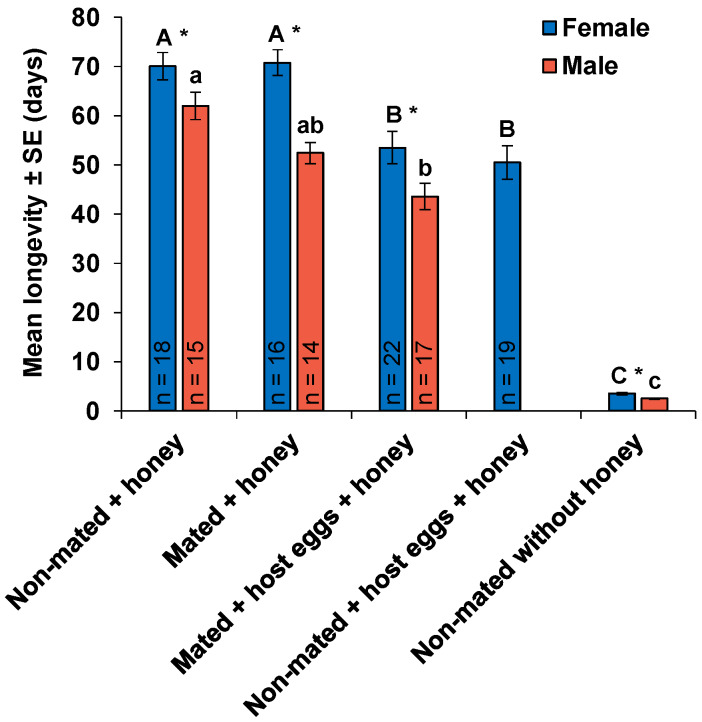
Mean (±SE) longevity of *Ooencyrtus lucidus* females and males in different treatments. n is the number of wasps that died naturally in each treatment excluding the ones that were stuck in honey. In the last treatment, non-mated without honey, all the initial number of wasps (n = 20) were used in the analyses. Significant differences are represented by capital letters for females and lower-case letters for males (Dunn’s Kruskal-Wallis multiple comparison, *p* < 0.05). The asterisks (*) represent significant differences between females and males within each treatment (Wilcoxon-Mann-Whitney test or *t*-test, *p* < 0.05).

Access to honey and host eggs had a significant effect on the longevity of *O. lucidus* females (*F*${}_{4,\;90}$ = 95.56, *p* < 0.001) (Figure 2). Mated and non-mated females fed with honey lived 70.8 ± 2.6 and 70.1 ± 2.8 days, respectively, and their longevity was not significantly different. Longevity of females that were provided with host eggs in addition to honey, and had reproductive activity significantly decreased by 24% (53.5 ± 3.3 days) in mated and by 28% (50.5 ± 3.4 days) in non-mated individuals. The effect of different treatments was also significant among males (*F*${}_{3,\;62}$ = 169.7, *p* < 0.001). Non-mated males provided with honey had the longest longevity of 62.0 ± 2.8 days, which was not significantly different from the longevity of mated males provided with honey (52.4 ± 2.2 days) but was significantly longer than longevity of males paired with ovipositing females (43.6 ± 2.7 days). Non-mated females and males deprived of honey only lived for 3.5 ± 0.2 and 2.5 ± 0.1 days after emergence, respectively (Figure 2). Females lived significantly longer than males in all treatments (treatment A (non-mated + honey): *Z* = 2.43, *P* < 0.05; treatment B (mated + honey): *t*${}_{28}$ = 5.35, *p* < 0.001; treatment C (mated + host eggs + honey): *t*${}_{37}$ = 2.20, *P* < 0.05; treatment E (non-mated without honey): *Z* = 4.02, *p* < 0.001).

**Figure 3:**
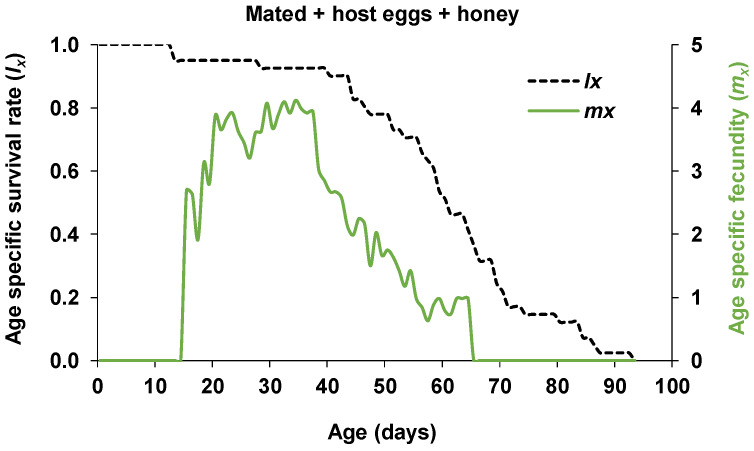
Age specific survival rate (dashed line) and fecundity (solid line) of mated *Ooencyrtus lucidus* on *Bagrada hilaris* eggs.

**Figure 4:**
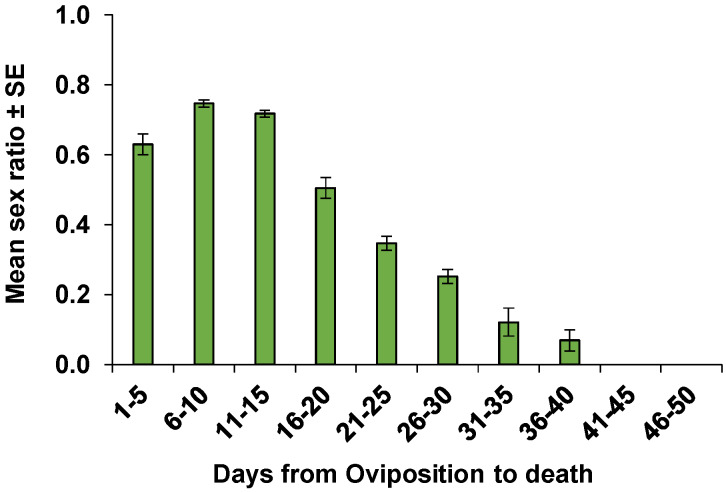
Mean (±SE) sex ratio (females/progeny/day) of mated females (n = 22) of *Ooencyrtus lucidus* on *Bagrada hilaris* eggs at five-day intervals from oviposition to death.

The pre-oviposition period of the mated females was on average 2.2 ± 0.2 days which was significantly longer than that of the non-mated females (1.5 ± 0.1 days) (*Z* = 2.75, *p* < 0.01) (Table 1). The first oviposition of mated females occurred on day 15 (2.7 eggs), and the daily fecundity increased to 3.9 eggs on day 20 and peaked on day 32 (4.1 eggs) (Figure 3). Mated females laid an average of 222.7 ± 12.6 eggs (ranged from 61 to 326) during their mean oviposition period of 37.3 ± 1.6 days, and the mean number of eggs laid daily by a female was 6.0 ± 0.3 eggs (Table 1). The mean fecundity of non-mated females was 217.9 ± 11.1 eggs (ranged from 107 to 290) during their mean oviposition period of 38.0 ± 1.8 days, and the mean number of eggs per female per day was 5.9 ± 0.4 eggs (Table 1). The number of eggs per day laid by a female ranged from 1 to 13 eggs (pedicels) for non-mated females and 1 to 15 eggs for mated females. When the number of pedicels exceeded the number of host eggs, superparasitism had occurred even though some of the eggs remained non-parasitized. The mean duration of the post-oviposition period was 13.9 ± 2.6 and 11.1 ± 2.4 days for mated and non-mated females, respectively. The average parasitism success rate which is the proportion of progeny emerged per eggs laid by a female per day was 0.77 ± 0.02 for the mated and 0.77 ± 0.03 for the non-mated females. The average sex ratio of progeny per mated female per day was 0.49 female progeny. Non-mated females produced only male progeny (Table 1). The average daily sex ratio of all 22 mated females (from emergence to death) for each 5-day interval is displayed in Figure 4. The average daily sex ratio was female biased from day 1 to 15 (ranging from 0.63 to 0.75), followed by a 1:1 ratio (0.50) from day 16 to 20. The average sex ratio then became male biased from day 21 to 40 (ranging from 0.35 to 0.07). In the last 10 days of their life (days 41-50), *O. lucidus* females produced only male progeny (Figure 4). The population growth parameters of mated *O. lucidus* were calculated as: net reproductive rate ($R_{0}$) = 103.8 females/female/generation, intrinsic rate of increase ($r_{m}$) = 0.171 female/female/day, the finite rate of increase (λ) = 1.187 population multiplication/day, the mean generation time (*T*) = 27.1 days, and the doubling time (*DT*) = 4.0 days (Table 2).

**Figure 5:**
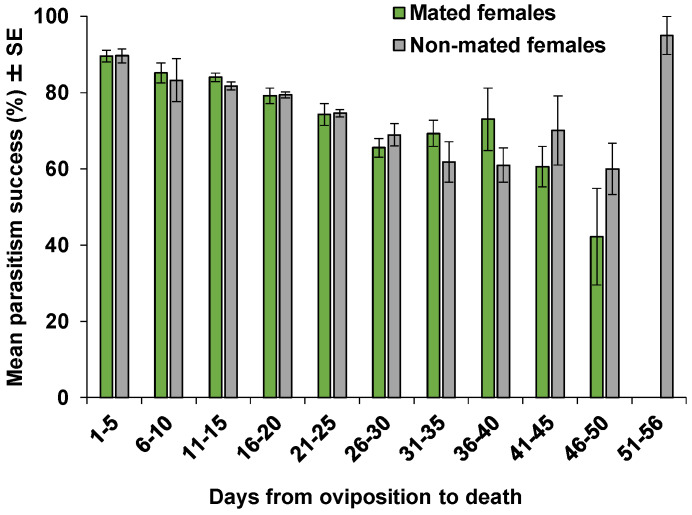
Parasitism success: mean (±SE) percentage of progeny emerged per eggs laid by all mated (n = 22) and non-mated (n = 19) females of *Ooencyrtus lucidus* on *Bagrada hilaris* eggs at five-day intervals from oviposition to death.

The average daily parasitism success rates indicate that more than 79% (ranging from 79.1% to 89.6%) of the parasitized eggs laid by mated females developed successfully when parasitism occurred in the first 20 days of females’ oviposition (Figure 5). From day 21 to 40, parasitism success rate ranged from 65.5% to 74.3%, and then declined to 60.6% from day 41 to 45, and 42.2% from day 46 to 50. For the non-mated females, parasitism success rate ranged from 79.4% to 89.6% from day 1 to 20 of the oviposition, and from 60.0% to 74.6% from day 21 to 50 of the oviposition. The high success rate from day 51 to 56 was due to the fact that only two females survived to these ages, and the emergence rate in eggs of those females was high (Figure 5).

**Figure 6:**
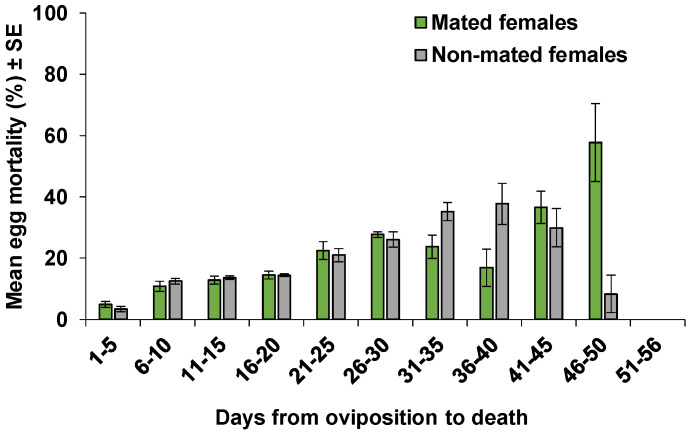
Mean (±SE) percentage of egg mortality in the progeny of all mated (n = 22) and non-mated (n = 19) females of *Ooencyrtus lucidus* on *Bagrada hilaris* eggs at five-day intervals from oviposition to death.

Of 4899 eggs laid by all mated females in treatment C, 911 eggs (18.6%) either failed to develop (86.3%) or developed but could not emerge from the host eggs (13.7%). Non-mated females parasitized 4140 eggs out of which 869 eggs (21%) died. For these females, mortality was 88.6% for the immature stages and 11.4% for the adults (Table 3). The majority of immature mortality occurred during the parasitoid egg stage, followed by pupal, and larval mortalities (Table 3). For the mated females, the average daily egg mortality increased from 5.0% to 27.7% in the first 30 days of females’ oviposition. From day 31 to 40, egg mortality declined to 16.9%, and then increased again to 57.8% from day 41 to 50 (Figure 6). For the non-mated females, the average egg mortality increased from 3.4% to 37.7% in the first 40 days of females’ oviposition, and then declined until it reached 0% in the last five days of oviposition (Figure 6). The low egg mortality from day 46 to 56 was due to the low number of females that survived to these ages and the high emergence rate in eggs of those females (Figure 5). 5.7% (52 out of 911 eggs) and 3.6% (31 out 869 eggs) of the eggs were superparasitized by the mated and non-mated females, respectively. The superparasitized eggs of mated females always yielded one offspring. However, 19% (6 out of 31 cases) of the superparasitized eggs of non-mated females yielded two males from the same egg.

## 4. Discussion

Understanding the life history of natural enemies is a fundamental step toward determining their possible success as biological control agents. Parasitoids, especially egg parasitoids, are of great importance to biological control since they kill their hosts before any damage is done [32,33]. The genus *Ooencyrtus* Ashmead includes more than 300 known species [34], most of which are egg parasitoids of major agricultural or forest pests from Lepidoptera and Heteroptera orders [35]. Some examples include *Ooencyrtus anasae* (Ashmead) [36,37], *Ooencyrtus fecundus* Ferriere & Voegele [38,39], *Ooencyrtus kuvanae* Howard [40,41], *Ooencyrtus nezarae* Ishii [42,43], *Ooencyrtus pistaciae* Hayat & Mehrnejad [44,45], *Ooencyrtus pityocampae* Mercet [46,47,48], *Ooencyrtus submetallicus* Howard [37], and *Ooencyrtus telenomicida* Vassiliev [49,50,51]. In this study, we determined biological characteristics of *O. lucidus*, a species that was found parasitizing *B. hilaris* eggs in the field ten years after the introduction of *B. hilaris* in the United States.

Our results indicate that, 14 days after oviposition, 93% of *O. lucidus* male and 69% of female progeny emerged, and the mean developmental time of males (14 days) was shorter than females (14.3 days) at 26 °C. The shorter developmental time of males allows them to have more mating events with newly emerging females and greater fertilization opportunities in those females. We observed that newly eclosed males remained close to the eggs from which they emerged. A similar behavior has been described for *Trichogramma* species (Hymenoptera: Trichogrammatidae) in which mating occurs at emergence sites [52,53]. Therefore, in the field, mating can occur on the host plant if early emerging males wait for females to emerge and mate with them. Moreover, as fecundity reaches a peak during the first week of emergence, it can be advantageous for males to be ready for mating during this period [54]. The developmental time of *O. lucidus* females on *B. hilaris* eggs is very close to that of *Ooencyrtus mirus* Triapitsyn & Power (14.5 days) on the same host at the same temperature [19]. Other species have shown longer developmental times on different hosts. For example, *O. telenomicida* females and males developed on *Graphosoma lineatum* L. (Hemiptera: Pentatomidae) in 15.8 and 15.4 days, respectively, at 26 °C [51]. *Ooencyrtus isabellae* Guerrieri & Noyes adults completed their development on *Zophiuma butawengi* (Heller) (Hemiptera: Lophopidae) in 15.5 days in ambient temperatures (25–30 °C) [55].

Adult longevity of parasitoids is a major factor affecting the field efficacy of parasitoids in biological control programs. Parasitoid performance can be enhanced by increased longevity, which leads to increased searching time and fecundity [56,57]. Longevity is significantly enhanced by food availability for adults, therefore provision of food sources like floral nectar, aphid honeydew or pollen is necessary to increase the parasitoids’ efficiency [55,58]. We obtained consistent results with *O. lucidus* as adults could survive over two months when provided with honey, and the longevity decreased significantly in honey-deprived adults. Therefore, access to a food source is necessary for *O. lucidus* survivorship in the environment, particularly when host populations are low. A study by Reference [59] also showed that *O. nezarae* adults can only live for 2–3 days after emergence without honey, but they can survive 40-60 days when provided with honey. These authors also indicated that the shortage of non-host food sources can negatively affect host searching behavior and oviposition of *O. nezarae*. Another study on *O. nezarae* indicated that females can only live for 2.5–3 days without honey, but access to honey increases their longevity to 31 days in the presence of host eggs and 23 days in the absence of host eggs [60]. In addition, *O. pityocampae* adults that were exposed to honey lived 10.5 times longer than those that did not receive it [61]. Similar results have been reported for *O. pityocampae* [46] and *O. isabellae* [55]. Our study indicates that host egg availability and reproductive activity reduces the longevity of *O. lucidus* females, suggesting a trade-off exists between fecundity and longevity. However, this negative correlation may disappear in optimal conditions when parasitoids can obtain enough resources from the environment to compensate for the energy drain due to reproduction [62].

We expect that *O. lucidus* is a synovigenic parasitoid like other *Ooencyrtus* species in which females emerge with no or few mature eggs, and egg maturation in the ovaries continues after emergence. Based on our results, *O. lucidus* requires a pre-oviposition period of 2.1 (ranged from 1 to 5) days in mated and 1.5 (ranged from 1 to 2) days in non-mated females. The significantly longer pre-oviposition period of the mated females compared to non-mated females can be due to the presence of males in the experimental vials in the former treatment since males could interfere with female oviposition. In mated *O. lucidus*, the maximum oviposition occurred between 7 and 24 days after emergence. In *O. kuvanae* [63] and *Ooencyrtus johnsoni* (Howard) [64], the number of eggs peaked 9–11 days after emergence. *Ooencyrtus lucidus* showed a very high average fecundity of 223 eggs per female at 26 °C. The fecundity of this parasitoid was much higher than that observed in other species, such as *O. pityocampae* (122 eggs at 27 °C on *Brachynema signatum* Jakovlev (Hemiptera: Pentatomidae)) [46], *O. kuvanae* (105 eggs on *Lymantria dispar* L. (Lepidoptera: Lymantriidae)) [65], and *O. nezarae* (75 eggs at 25 °C and 54 eggs at 27 °C on *Riptortus clavatus* (Thunberg) (Hemiptera: Alydidae)) [66]. The population growth parameters of *O. lucidus* also were superior to other species, such as *O. telenomicida* [51]. The net reproductive rate of *O. lucidus* (103.8 females/female/generation) on *B. hilaris* eggs was 2 times the potential of *O. telenomicida* on *G. lineatum* eggs (53.0 females/female/generation). This contributes to a higher intrinsic rate of increase (0.171 versus 0.154 female/female/day), and shorter doubling times (4.0 versus 4.5 days) for *O. lucidus* than *O. telenomicida* [51].

The sex ratio of *O. lucidus* progeny decreased with the age of the mother. Regulation of sex allocation with maternal age has been reported for other parasitoids [67,68,69]. Several physiological factors can result in the sex change and increase of male production by aged females, such as sperm depletion or senescence, reduced sperm viability, or weakened control of sperm release from spermatheca [70,71,72,73]. Sex ratio can also be affected by developmental mortality [74,75], superparasitism [76,77], or *Wolbachia* infection [78]. Since the daily sex ratio is the number of females produced each day as a proportion of the daily fecundity, a decrease in the number of female progeny or a decrease in fecundity can decrease the progeny sex ratio. Fecundity of mated *O. lucidus* females that were over 40 days old declined, and only male progeny were produced. Therefore, both decreased fecundity and sperm depletion could contribute to the sex ratio change of progeny as maternal age increased. Furthermore, the males in the present study were not replaced during the experiment, therefore, a high frequency of mating with one male could lead to a decrease in sperm numbers or sperm depletion [79].

The emergence rate of 95.2% was obtained from eggs with one pedicel (non-superparasitized eggs) laid by 3-day-old parent wasps. The average parasitism success rate in the F${}_{2}$ generation in the first five days of oviposition was 89.6% (ranging from 84.2% to 92.9%). The lower success rate in the F${}_{2}$ generation is due to the fact that we utilized all eggs, including the superparasitized ones, even though there were less than 6%. This low superparasitism rate may be caused by an ability to discriminate between parasitized and non-parasitized eggs. Studies have confirmed that the parasitoid’s egg pedicel serves as an external marker and is responsible for host discrimination after parasitism in several *Ooencyrtus* species, including *O. nezarae* [80] and *O. pityocampae* [81]. Whether this exists for *O. lucidus* needs to be tested through behavioral observations. Another factor that is known to contribute to immature mortality is reduced viability of females’ eggs over time [82,83], which we documented in the F${}_{2}$ generation.

Interestingly, we observed more progeny survival in the superparasitized eggs laid by non-mated females compared to mated females. Since the progeny of non-mated females were all males which have smaller body sizes, it suggests that the nutritional resources of a *B. hilaris* egg are sufficient enough only for the survival of two males, and not for two females or one female and one male.

The results obtained in this study suggest that *O. lucidus* has the basic biological parameters that are desired for *B. hilaris* management. However, a parasitoid’s reproductive capacity in the field can be restricted by the host’s density and its accessibility [84]. *Bagrada hilaris* eggs are laid individually or in very small clusters of 2 to 3 eggs on plants or in the soil [3,85]. Therefore, there might be a trade-off between the energy required to locate and parasitize these individual hidden eggs and fecundity [86]. Further field studies are needed to better understand how effective this parasitoid species would be for the biological control of *B. hilaris*.

## Figures and Tables

**Table 1:** Reproduction parameters (mean ± SE) of *Ooencyrtus lucidus* on *Bagrada hilaris*.

Parameters	Mated Females	Non-Mated Females	*p*
Pre-oviposition period (days)	$2.2\pm 0.2{\;}^{a}$	$1.5\pm 0.1{\;}^{b}$	0.0066
Oviposition period (days)	$37.3\pm 1.6{\;}^{a}$	$38.0\pm 1.8{\;}^{a}$	0.7819 *
Post-oviposition period (days)	$13.9\pm 2.6{\;}^{a}$	$11.1\pm 2.4{\;}^{a}$	0.5037
Total fecundity (eggs/female)	$222.7\pm 12.6{\;}^{a}$	$217.9\pm 11.1{\;}^{a}$	0.7807
Fecundity rate (eggs/female/day)	$6.0\pm 0.3{\;}^{a}$	$5.9\pm 0.4{\;}^{a}$	0.8614 *
Parasitism success rate (progeny/eggs laid by a female/day)	$0.77\pm 0.02{\;}^{a}$	$0.77\pm 0.03{\;}^{a}$	0.7120
Sex ratio ([females/progeny]/female/day)	0.49±0.04	0.00 (all male)	

Values within the same row followed by the same letter are not significantly different, Wilcoxon-Mann-Whitney test or * *t*-test (*p* < 0.05).

**Table 2:** Population growth parameters (mean ± SE) of *Ooencyrtus lucidus* on *Bagrada hilaris* eggs.

Parameters	Equations	Mated Females
Net reproductive rate ($R_{0}$)	$\sum l_{x}m_{x}$	103.8±12.0
Intrinsic rate of increase ($r_{m}$)	$\sum {}_{x=0}^{n}e^{-rx}l_{x}m_{x}\;=\;1$	0.171±0.005
Finite rate of increase (λ)	$e^{r_{m}}$	1.187±0.005
Mean generation time (*T*)	lnR0rm	27.1±1.1
Doubling time (*DT*)	ln2rm	4.0±0.1

**Table 3:** Parasitism failure: percentage of mortality during development of *Ooencyrtus lucidus* on *Bagrada hilaris* eggs.

Life Stages	Mated Females			Non-Mated Females			*X* ${}^{2}$	*p*
	Number of Dead	Number of Dead at each Stage	Mortality (%)	Number of Dead	Number of Dead at each Stage	Mortality (%)		
Eggs	911	716	78.6	869	679	78.1	0.06	0.7980
Larvae		20	2.2		38	4.4	6.79	0.0091 *
Pupae		50	5.5		53	6.1	0.29	0.5882
Adults		125	13.7		99	11.4	2.14	0.1436

The asterisks (*) represent significant differences in mortality percentages between mated and non-mated females (Chi-squared test, *p* < 0.05).
